# The Frequency and Clinical Relevance of Multidrug Resistance Protein Expression in Patients with Lymphoma

**DOI:** 10.5505/tjh.2012.60362

**Published:** 2012-06-05

**Authors:** Eren Gündüz, Murat Dinçer, Güniz Yıldız, Cengiz Bal, Zafer Gülbaş

**Affiliations:** 1 Eskişehir Osmangazi University, Department of Hematology, Eskişehir, Turkey; 2 Eskişehir Acıbadem Hospital, Medical Oncology Clinic, Eskişehir, Turkey; 3 Eskişehir Osmangazi University, Department of Biostatistics, Eskişehir, Turkey; 4 Anadolu Health Center in Affiliation with Johns Hopkins Medicine, Bone Marrow Transplantation Center, Kocaeli, Turkey

**Keywords:** Multidrug resistance, non-Hodgkin’s lymphoma, Hodgkin’s lymphoma, Survival

## Abstract

**Objective:** Multidrug resistance is a cause of treatment failure in patients with malignant lymphoma; however, the frequency and clinical relevance of multidrug resistance protein expression are unclear. The present study aimed to investigate expression of the most common multidrug resistance proteins in a group of lymphoma patients.

**Material and Methods: **The study included 44 previously untreated lymphoma patients (non-Hodgkin’s lymphoma [n = 21], non-malignant lymphadenopathy [n = 13], and Hodgkin’s lymphoma [n = 10]). MDR1, MRP, and LRP expression was assessed via quantitative PCR of lymph node biopsy specimens.

**Results: **In the non-Hodgkin’s lymphoma group MDR1 was positive in 23.8% (5/21) of the patients, MRP was positive in 57.14% (12/21), and LRP was positive in 90.47% (19/21). In the non-malignant lymphadenopathy group, MDR1 was positive in 46.15% (6/13) of the patients, MRP was positive in 84.61% (11/13), and LRP was positive in 100% (13/13). In the Hodgkin’s lymphoma group MDR1 was positive in 50% (5/10) of the patients, MRP was positive in 50% (5/10), and LRP was positive in 80% (8/10). MDR1, MRP, and LRP expression did not differ between the 3 groups. Furthermore, MDR1, MRP, and LRP expression wasn’t associated with tumor stage, response to first-line therapy, the erythrocyte sedimentation rate, or C reactive protein, beta 2 microglobulin, serum lactate dehydrogenase, and albumin levels. Additionally, survival time in the MDR1- and MRP-positive, and MDR1- and MRP-negative patients did not differ (comparison of LRP was not possible due to the small number of LRP-negative patients).

**Conclusion:** According to the present findings, future studies should investigate alternative pathways of multidrug resistance in order to arrive at a better understanding of treatment failure in lymphoma patients.

## Abstract (tr)

**Amaç:** Çoklu ilaç direnci malign lenfomalarda tedavi başarısızlığının nedenlerindendir. Fakat çoklu ilaç direnci proteinlerinin sıklığı ve klinik önemiyle ilgili çelişkiler mevcuttur. Biz bu çalışmada lenfomalı hastalarımızda en sık görülen çoklu ilaç direnci proteinlerinin ifadelerini araştırdık.

**Gereç ve Yöntemler:** Çalışmaya daha önce tedavi almamış 44 hasta (21 non Hodgkin lenfoma, 13 malign olmayan lenfadenopati, 10 Hodgkin lenfoma) alındı. MDR1, MRP ve LRP ifadeleri lenf nodu biyopsi örneklerinde kantitatif PCR ile değerlendirildi.

**Bulgular:** Non Hodgkin lenfomada MDR1 %23.8 (5/21), MRP %57.14 (12/21), LRP %90.47 (19/21) pozitifti. Malign olmayan lenfadenopatili hastalarda MDR1 %46.15 (6/13), MRP %84.61 (11/13), LRP %100 (13/13) pozitifti. Hodgkin

lenfomada MDR1 %50 (5/10), MRP %50 (5/10), LRP %80 (8/10) pozitifti. MDR1, MRP ve LRP ifadeleri 3 grup arasında farklı değildi. İfadeler tümör evresi, eritrosit sedimantasyon hızı, C reaktif protein, beta 2 mikroglobulin, ilk sıra tedaviye yanıt, serum laktat dehidrogenaz ve albumin düzeyiyle ilişkili değildi. MDR1 ve MRP pozitif ve negatif hastaların yaşam süreleri arasında da fark bulamadık (LRP pozitif ve negatif hastalar arası karşılaştırma yapmak az hasta sayısı nedeniyle mümkün olmadı).

**Sonuç:** Verilerimize göre, bu grup hastalardaki tedavi başarısızlığını aydınlatmak için gelecekte yapılacak çalışmalar çoklu ilaç direncinin alternatif yolaklarına yönlendirilebilir.

## INTRODUCTION

Multidrug resistance (MDR) refers to the resistance oftumor cells to chemotherapeutic agents of varying chemicalstructure and mechanisms of action [1]. Numerousmechanisms are involved in drug resistance; among them,drug efflux transporters are one of the most intensivelystudied and most prevalent [2,3]. Common MDR proteinsinclude permeability related glycoprotein (P-gp),multidrug resistance-associated protein (MRP), and lungresistance-related protein (LRP) [4,5]. P-gp, also referredto as P-170, is a product of the MDR1 gene and is an ATPdependentpump capable of expelling drugs from cancercells [6]. MRP is structurally similar to P-gp and is a memberof the same transmembrane transporter superfamily[7]. LRP is a 110-kDa protein identified in a P-gp-negativeMDR lung cancer cell line and functions as a major vaultprotein in humans [8].Overexpression of MDR increases efflux of most lymphomaregimens from cells [9,10,11]. The predictive andprognostic value of MDR expression has previously beenreported for multiple myeloma (MM) [12], acute myeloidleukemia (AML) [13], acute lymphoblastic leukemia (ALL)[14], and adult T-cell leukemia [15]. The MDR phenotypeis also the major cause of treatment failure in patientswith malignant lymphoma; however, findings regardingexpression of the MDR1 gene/P-gp in malignant lymphomapatients are inconsistent [16,17,18].Due to the non-clarity surrounding the frequency andclinical relevance of multidrug resistance protein expression,the present study aimed to investigate the expressionof the most common MDR proteins in a group of previouslyuntreated patients with lymphoma—specifically,whether or not these 3 multidrug resistance proteins wereexpressed and their impact on clinical outcome.

## MATERIALS AND METHODS

The study included 44 previously untreated patientsthat were diagnosed between 2005 and 2007. The patientswere divided into 3 groups, according to pathology results.Group 1 included 21 patients diagnosed a diffuse largeB-cell lymphoma (DLBCL) (n=9), T-cell non-Hodgkin’slymphoma (NHL) (n=8), and mantle cell lymphoma(MCL) (n = 4). Group 2 included 13 patients diagnosed asreactive lymphadenopathy (LAP) (n=5), granulomatousinflammation (n = 5), dermatopathic LAP (n = 1), benignmixed tumor (n=1) and Kikuchi’s disease (n=1). Group3 included 10 patients diagnosed as Hodgkin’s lymphoma(HL).

Survival time was defined as the period (months)from diagnosis to death or data analysis. MDR1, MRP,and LRP expression was assessed via quantitative PCR oflymph node biopsy specimens. Only biopsy specimensclearly proven to be malignant based on flow cytometricand pathological analysis were included in the study.Peripheral blood and lymph node biopsy specimenswere obtained following provision of informed consentby the patients and approval of the study protocol bythe Eskişehir Osmangazi University, School of MedicineInstitutional Review Board.

The patients were treated with CVP, CHOP, Hyper-CVAD, IMVP16, DHAP, ESHAP, ABVD, and BEACOPPpolychemotherapy. Patients with DLBCL and MCL alsoreceived rituximab. In all, 4 patients required radiotherapyand 4 other patients received additional peripheralstem cell transplantation (3 autologous and 1 allogeneicwith reduced intensity conditioning). Response to chemotherapywas assessed according to standard criteria [19].Tumor stage, the erythrocyte sedimentation rate, and Creactive protein, beta 2 microglobulin, serum LDH, andalbumin levels were also recorded.

**Detection of multidrug resistance**

Lymph node biopsy specimens obtained in the surgicalsuite were immediately taken to the laboratory in Eppendorftubes containing RPMI1640 medium. The specimenswere preserved in liquid nitrogen at –80 °C and thawedat 4 °C before RNA isolation. An mRNA Isolation Kit II(Tissue) (Roche) was used to obtain RNA from the tissuesamples using a MagNA Pure instrument. cDNA wasobtained using a Transcriptor First Strand cDNA SynthesisKit (Roche). MDR1, MRP, LRP, and beta actin primaryprobes from TIB MOLBIOL and Light Cycler TaqmanMaster reaction mix were used for quantitative PCR in aRoche Light Cycler instrument. As beta actin is presentin all clinical samples, it was used as an intrinsic control.Quantitative MDR1, MRP, and LRP values were dividedby the beta actin value, and the positive results were usedfor statistical analysis. We did not set a cut-off value andconsidered any level of expression as positive.

**Statistical analysis**

All statistical analyses were performed using SPSS(PASW) v.18.0 for Windows software. Distribution of thevariables was determined using the Shapiro Wilks test.Parametric tests were used to analyze normally distributeddata and non-parametric tests were used for data not normallydistributed. The chi-square test was used for analysisof cross tables. Correlations between variables weredetermined by calculating Spearman’s correlation coefficients.The independent samples t-test and ANOVA wereused to compare group means of normally distributedvariables. Tukey’s post hoc test was used to determinedifferent groups in ANOVA. The Kaplan Meier test wasused to compare survival time between ≥2 groups. Thelog rank test was used to determine differences betweenmean survival times. P<0.05 was considered statisticallysignificant. Data are expressed as mean ± standard deviation(SD).

## RESULTS

Mean age was 53.2±17.5 years in the NHL patients(group 1), 41.7 ± 15.1 years in the non-malignant LAPpatients (group 2), and 37.6±13.7 years in the HL patients(group 3). NHL patients were older than HL patients (P< 0.05); the difference was not significant. Mean survivaltime was 19.69±4.33 months in group 1, 42.46±5.85months in group 2, and 49.50±4.69 months in group 3.NHL patients had shorter survival than HL patients (P <0.01), but survival time did not differ between the reactiveLAP and lymphoma patients (P > 0.05) (Figure).MDR1, MRP, and LRP positivity in the 3 groups isshown in the Table. The frequency of MDR1, MRP, andLRP positivity was significantly different between the 3groups (P > 0.05). While examining survival time in eachgroup separately, comparison was not possible in somegroups due to the small number of negative patients (MRP and LRP in group 2, and LRP in groups 1 and 3). When theother groups were compared (MDR1 and MRP in group 1and 3, and MDR1 in group 2) a significant difference insurvival time between the MDR1- and MRP-positive, andthe MDR1- and MRP-negative patients was not observed.In all, 5 patients in group 1 were positive for all 3 MDRproteins, 7 were positive for MRP and LRP, and 7 werepositive only for LRP. In total, 6 patients in group 2 werepositive for all 3 MDR proteins, 5 were positive for MRPand LRP, and 2 were positive only for LRP. Positivity forall 3 MDR proteins was observed in 3 patients in group 3patients, whereas 1 patient was positive for MRP and LRP,1 patient was positive only for MDR1, and 3 patients werepositive only for LRP. There weren’t any significant associationsbetween MDR1, MRP, and LRP expression, andtumor stage, response to first-line therapy, the erythrocytesedimentation rate, or C reactive protein, beta 2 microglobulin,serum LDH and serum albumin levels.

## DISCUSSION

In the present study expression of MDR1, MRP, andLRP was determined via quantitative real-time PCR (RTPCR)in patients with lymphoma and non-malignant diseases,and the association between the expression of the3 MDR proteins, and clinical and laboratory parameterswas evaluated. To the best of our knowledge this is thefirst study to evaluate the frequency and clinical relevanceof MDR proteins in patients with malignant lymphomas(both Hodgkin’s and non-Hodgkin’s) and non-malignantdiseases. In contrast to AML, studies on lymphomas andMDR are few in number and have generally focused onP-gp expression, and most included a heterogeneouspatient population. Furthermore, the methods used todetermine MDR expression in such studies varied widely[20]. As such, comparison of the published data is difficult.Moreover, these studies included a small number ofpatients, as did the present study, and therefore definitiveconclusions cannot be reached.

MDR1/P-gp expression in lymphomas has been previouslyexamined. Liu et al. [21] reported that 41.7%of 24 B-cell lymphoma patients were MDR-1/P-gp-positive(based on RT-PCR) before treatment. Pileri et al.[22] reported that 44% of peripheral T-cell lymphomapatients and 40% of B-cell lymphoma patients were P-gppositivebefore treatment. Findings regarding the clinicalimportance of MDR1/P-gp in lymphomas are inconsistent.MDR1/P-gp was reported to be predictive of a poorresponse to induction chemotherapy in 2 studies [22,23],but not in 2 other studies [24,25]. Overall, research showsthat 2%-30% of lymphomas express P-gp immunohistochemically [22,23,24,26], increasing to 22%-50% whenRNA-based analysis methods for detecting P-gp/MDR1 areused [24,25,27]. MDR1 expression in the present studywas observed in 23.8% of non-Hodgkin’s lymphomapatients and in 50% of Hodgkin’s lymphoma patients;these percentages are similar to those previously reported.The present study’s findings are also in agreement withstudies that did observe a relationship between MDR1expression [24,25] and poor response to induction chemotherapy.

Filipits et al. [28] reported that LRP was positive in23% and MRP1 was positive in 44% of newly diagnosedDLBCL patients. LRP expression was associated with poorresponse to chemotherapy and shorter survival, whichsuggests that LRP is a clinically relevant drug resistancefactor in DLBCL. A similar predictive and prognostic valueof LRP expression was reported in patients with AML[15,29], ALL [30], MM [12], and advanced ovarian cancer[11]. Ohsawa et al. [31] reported that MRP1 was positivein 63% and LRP was positive in 68% of patients withnodal DLBCL. Huang et al. [32] observed MRP and LRPexpression rates of 20.5% and 12.5%, respectively, in nasalNK/T-cell lymphoma patients. It is likely that the expressionof MDR varies according to lymphoma subtype. In thepresent study expression of MRP and LRP was observed in 57.14% and 90.47% of NHL patients, respectively (LRP expression was higher than previously reported in Filipits[28], Oshawa [31] and Huang’s [32] studies), versus MRP and LRP in 50% of HL patients, respectively.

MRP expression was not observed to play an importantrole in the mechanism of drug resistance associated with apoor clinical outcome in previously untreated NHL patients[33]. Filipits et al. [28] reported that MRP1 expression hadno impact on the outcome of chemotherapy or survivalin patients with DLBCL, and similar findings have beenobserved in refractory lymphoma patients in whom MRP expression was not determined via quantitative PCR [34].Previous studies on AML [14,29], ALL [30], and advancedovarian cancer [11] also failed to observe any predictiveor prognostic significance of MRP1 expression. Additionally,in the present study MRP and LRP expressions had nocorrelation with response to induction chemotherapy orsurvival in lymphoma patients with mixed histopathology.

In the present study there weren’t any significant correlations between MDR1, MRP, and LRP expression, andtumor stage, response to first-line therapy, the erythrocytesedimentation rate, or C reactive protein, beta 2 microglobulin,and serum LDH and albumin levels in the NHLand HL patients. Mixed histopathology and differences in treatment between the patients make it impossible to correlate the findings with clinical outcome, which is a limitationof the present study that warrants additional research. Anti-CD20 monoclonal antibody (rituximab) has becomethe standard therapy for aggressive NHL and has dramaticallyimproved treatment outcome [35]. Recent laboratory-based evidence shows that rituximab interacts withP-gp [36]; unfortunately, due to mixed histopathologyand the small number of patients (n = 13) in the presentstudy, we were unable to examine that interaction.

Non-malignant tissues expressed MDR proteins in thepresent study and the frequency of expression was similarin all the lymphoma patients. In fact, normal lymphocyteswere reported to express MDR1/P-gp [37], but Kang etal. [9] posited that contamination of tumor samples withT-lymphocytes and monocytes does not significantlyincrease the level of MDR1 expression. The similarity of MDR expression between lymphoma and reactive LAP patients observed in the present study may have beendue to lack of a cut-off value and considering any level of expression as positive, or that 3 patients previouslyreported as reactive LAP were diagnosed as NHL followingrebiopsies performed during follow-up.

In conclusion, MDR1, MRP, and LRP expression wasnot observed to influence overall survival in NHL andHL patients. The present findings indicate that additionalstudies should examine alternative pathways of MDRother than MDR1/P-gp, MRP and LRP expression (apoptosisetc.) to elucidate treatment failure in this group ofpatients. Furthermore, the frequencies of expression observed in the present study are noteworthy, as to thebest of our knowledge this is the first study to report andcompare these frequencies in HL patients and non-malignant patients.

**Conflict of Interest Statement**

The authors of this paper have no conflicts of interest,including specific financial interests, relationships, and/or affiliations relevant to the subject matter or materialsincluded.

## Figures and Tables

**Table 1:**
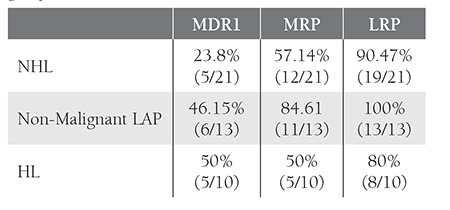
MDR1, MRP, and LRP positivity in the non-Hodgkin’slymphoma, non-malignant LAP, and Hodgkin’s lymphomagroups

**Figure 1:**
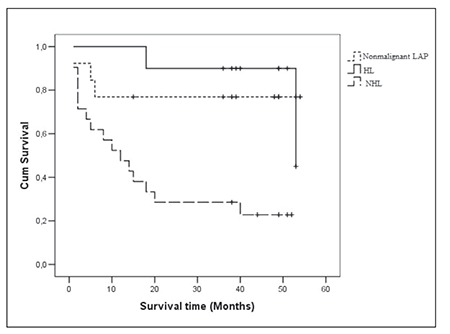
Survival time in the non-Hodgkin’s lymphoma,non-malignant LAP, and Hodgkin’s lymphoma groups
